# Cystic cardiac myxoma: a rare variant of cardiac myxoma in a woman with acute stroke

**DOI:** 10.1186/s43044-024-00571-6

**Published:** 2024-10-10

**Authors:** Damandeep Singh, Priya Jagia, Aprateem Mukherjee, Alaina Zameer, Sudheer Kumar Arava

**Affiliations:** 1 Department of Cardiovascular Radiology and Endovascular Interventions, All India Institute of Medical Sciences, New Delhi, 110029 India; 2 Department of Cardiology, All India Institute of Medical Sciences, New Delhi, 110029 India; 3 Department of Pathology, All India Institute of Medical Sciences, New Delhi, 110029 India

**Keywords:** Cystic cardiac mass, Myxoma, Cardiac magnetic resonance imaging, Left atrium

## Abstract

**Background:**

Cardiac myxomas are the most common primary benign tumors of the heart usually presenting as an oval mass attached to the interatrial septum. Mild heterogenous enhancement is typically seen upon contrast administration. Myxomas are benign, however can present with embolic episodes necessitating prompt diagnosis and treatment.

**Case presentation:**

A middle-aged woman presenting with acute ischemic stroke was detected to have a complex cystic mass in the left atrium on echocardiography. Further evaluation with cardiac magnetic resonance imaging narrowed a differential diagnosis to hemangioma, hemangioendothelioma, sarcoma, hydatid cysts, bronchogenic cysts, and cystic variant of cardiac myxoma. CTA demonstrated arterial supply from the left circumflex artery supplying the lesion. Following surgical excision, histopathology analysis suggested a cystic cardiac myxoma with secondary degeneration.

**Conclusion:**

Cystic masses in the heart are challenging to diagnose due to similar imaging characteristics, and misdiagnosis may lead to further major downstream complications such as stroke, hemorrhage, and hemodynamic alterations causing syncope. Therefore, surgical excision of a cystic cardiac mass is justified to establish a definitive histopathological diagnosis and prevent further downstream complications. We hereby report a rare case of cystic cardiac myxoma in the left atrium showing intense progressive enhancement in CMR and CTA in a middle-aged woman presenting with acute stroke. This case highlights an exceedingly rare variant of cystic cardiac myxoma.

**Supplementary Information:**

The online version contains supplementary material available at 10.1186/s43044-024-00571-6.

## Background

Cardiac myxomas are the most common primary benign tumors of the heart usually seen as solid round-to-oval masses attached to interatrial septum (IAS) and arising from mesenchymal cells of septal endocardium. It is composed of myxoid matrix, which leads to its gelatinous appearance [1]. Cardiac myxomas are seen in 0.03% of the general population with an estimated annual incidence of 0.5–1 case per million individuals [2, 3]. Myxomas occurring in left-sided heart chambers can embolize to cerebral and peripheral circulation, whereas right-sided myxomas can lead to pulmonary embolism [4]. The variable composition of myxoid tissue, fibrous component, blood, and hemorrhagic breakdown products within myxomas results in significant variability of the signal characteristics on cardiac magnetic resonance imaging (CMR). Myxomas usually show mild heterogenous enhancement on contrast administration with area of enhancement corresponding to inflammation and rich myxomatous element [5, 6]. Cystic myxomas in left atrium (LA) are exceedingly rare with only a few case reports described in the literature [7–10]. The differential diagnosis of cystic masses in LA can be hydatid cyst, IAS aneurysm, coronary artery aneurysm, intramural hematoma of LA, sarcomas, hemangiomas, hemangioendotheliomas, bronchogenic cysts and rarely myxomas [11–15].

## Case presentation

**Fig. 1:**
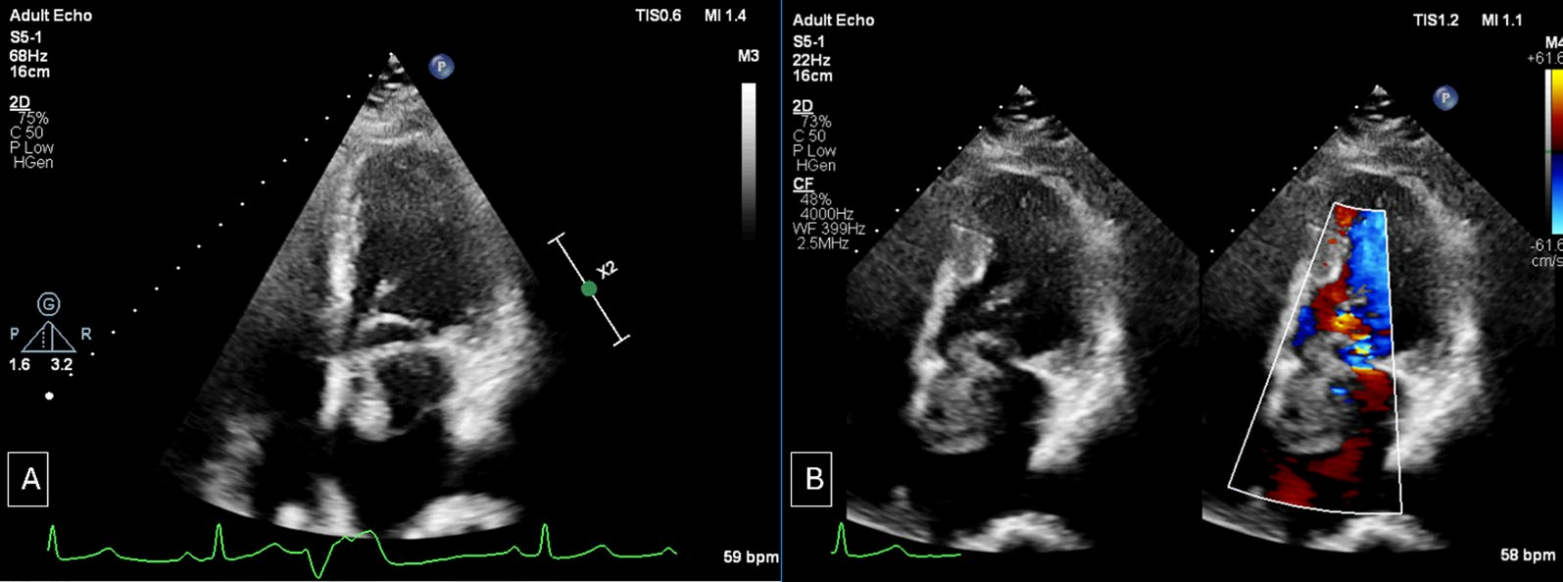
Apical four-chamber view demonstrating complex solid cystic mass lesion in left atrium protruding across the mitral annulus (**A**) causing dynamic transmitral inflow stenosis during ventricular diastole (**B**)

**Fig. 2:**
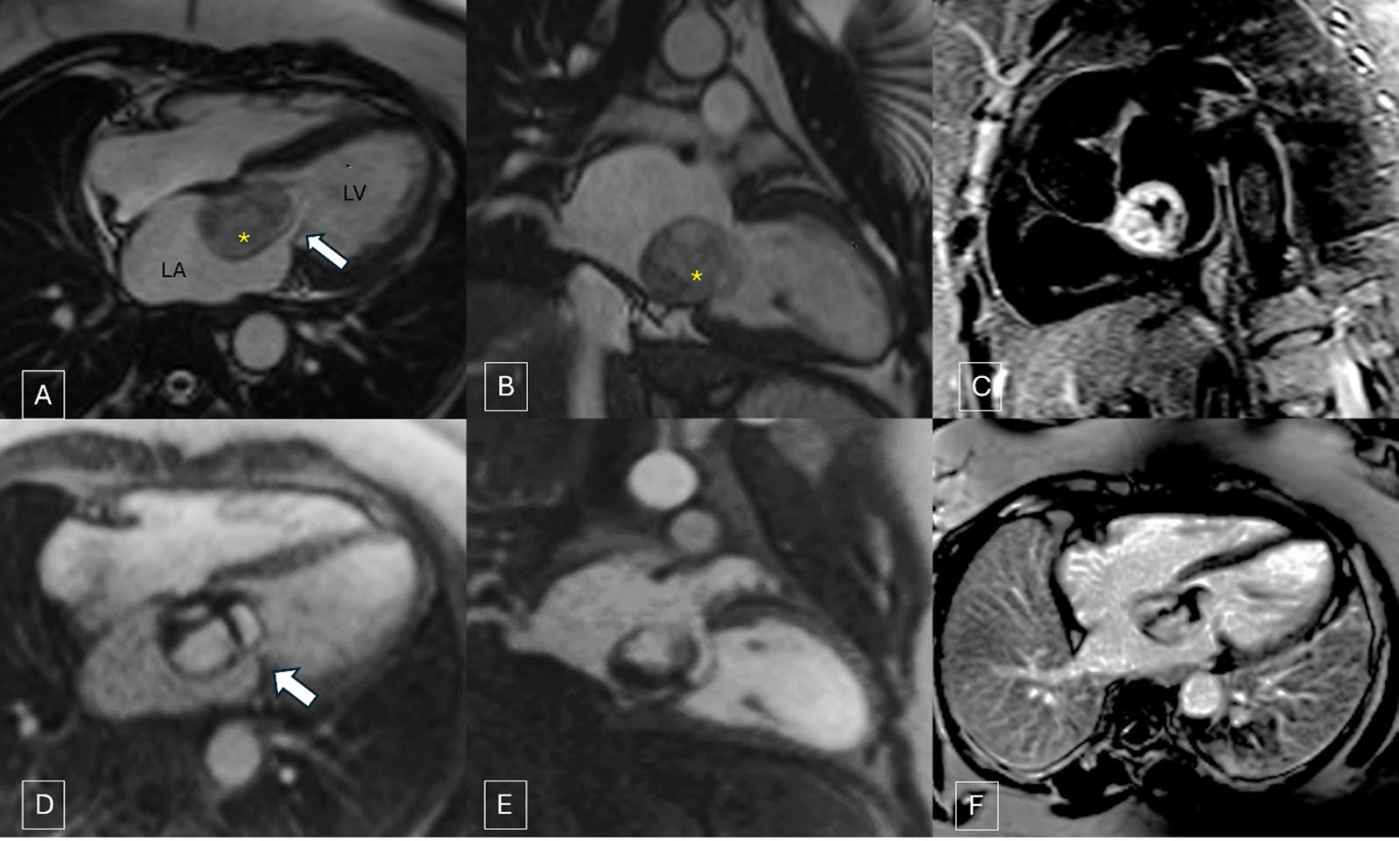
CINE steady-state free precession (SSFP) horizontal long axis (**A**) and vertical long-axis (**B**) images showing heterogeneous hyperintense mass (yellow asterisk) with intrinsic hypointense bands seen in the left atrium attached to inferior aspect of IAS protruding across the mitral annulus (white arrow). The solid component of mass is hyperintense with suppressed signal from cystic spaces. (**C**) On dynamic first pass perfusion (**D, E**) intense progressive enhancement seen within the cystic part of the lesion with non-enhancing hypointense solid component. On LGE images, persistent enhancement of cystic spaces was seen without enhancement in solid component (**F**)

A 53-year-old woman who presented to emergency with right-sided hemiparesis underwent non-contrast computed tomography brain which showed an acute infarct in the left thalamic and gangliocapsular region. She was administered recombinant tissue plasminogen activator. She did not have any relevant past medical or surgical history. Therefore, as part of screening workup she underwent a transthoracic echocardiography which detected a complex cystic mass in the LA attached to the IAS dynamically protruding across mitral annulus into left ventricle (LV) which led to suspicion of a hydatid cyst (Fig. 1). Subsequently, a contrast-enhanced CMR was done which showed a well-defined pedunculated solid cystic mass measuring approximately 4 cm × 3 cm in LA, attached to the inferior aspect of IAS and prolapsing through mitral valve during diastole causing dynamic transmitral inflow stenosis (Supplementary clip 1). The cystic areas within mass appeared hyperintense on T2-weighted images (T2WI) and hypointense on T1-weighted images (T1WI), whereas solid component appeared hypointense on both T1WI and T2WI. On the first pass perfusion, intense progressive enhancement was seen within the cystic part of the lesion immediately following the left-sided cardiac chamber enhancement. The solid component did not show any discernible enhancement (Supplementary clip 2). On late gadolinium enhancement (LGE) sequences, persistent enhancement of cystic spaces was seen without any enhancement in solid component (Fig. 2). High inversion time (TI) images did not show any thrombus. Biventricular functions were normal with no abnormal signal intensity seen in the myocardium. On the basis of CMR, possible differential diagnosis of hemangioma, hemangioendothelioma and cystic variant of myxoma was given.

**Fig. 3:**
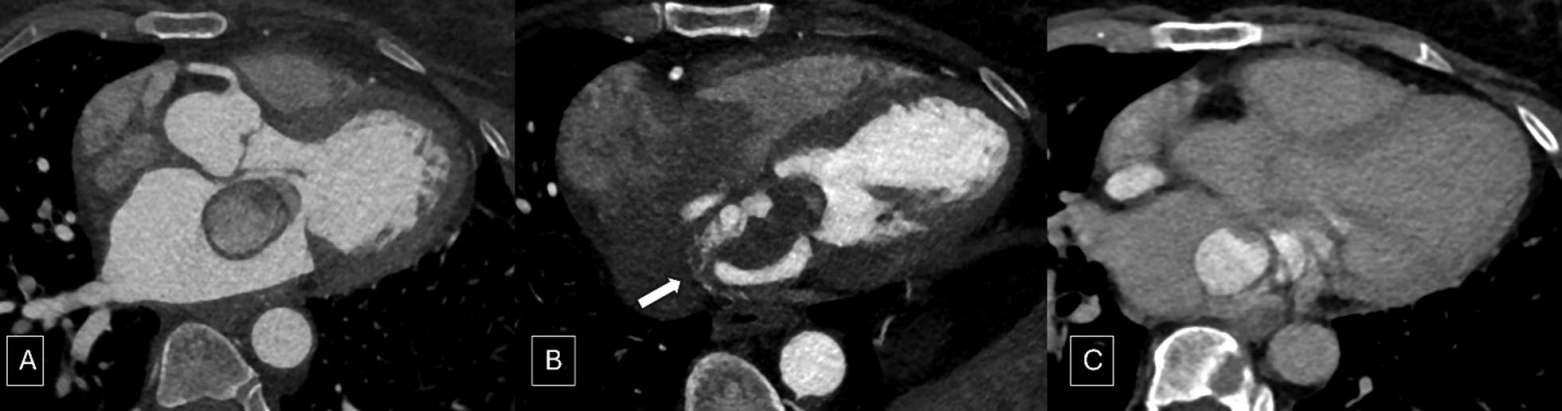
Cardiac CTA showing solid cystic enhancing mass lesion supplied from branches of left circumflex artery (**A, B**). On delayed images, progressive pooling of contrast was seen within cystic spaces

Patient was planned for surgical excision of the mass, and a cardiac CTA was done for evaluation of coronary artery disease. CTA confirmed the findings of CMR and additionally showed multiple arteries from branches of the left circumflex artery. Progressive enhancement of cystic spaces was seen on CTA with non-enhancing solid components. No calcification was seen. No obstructive coronary artery disease was noted (Fig. 3).

**Fig. 4:**
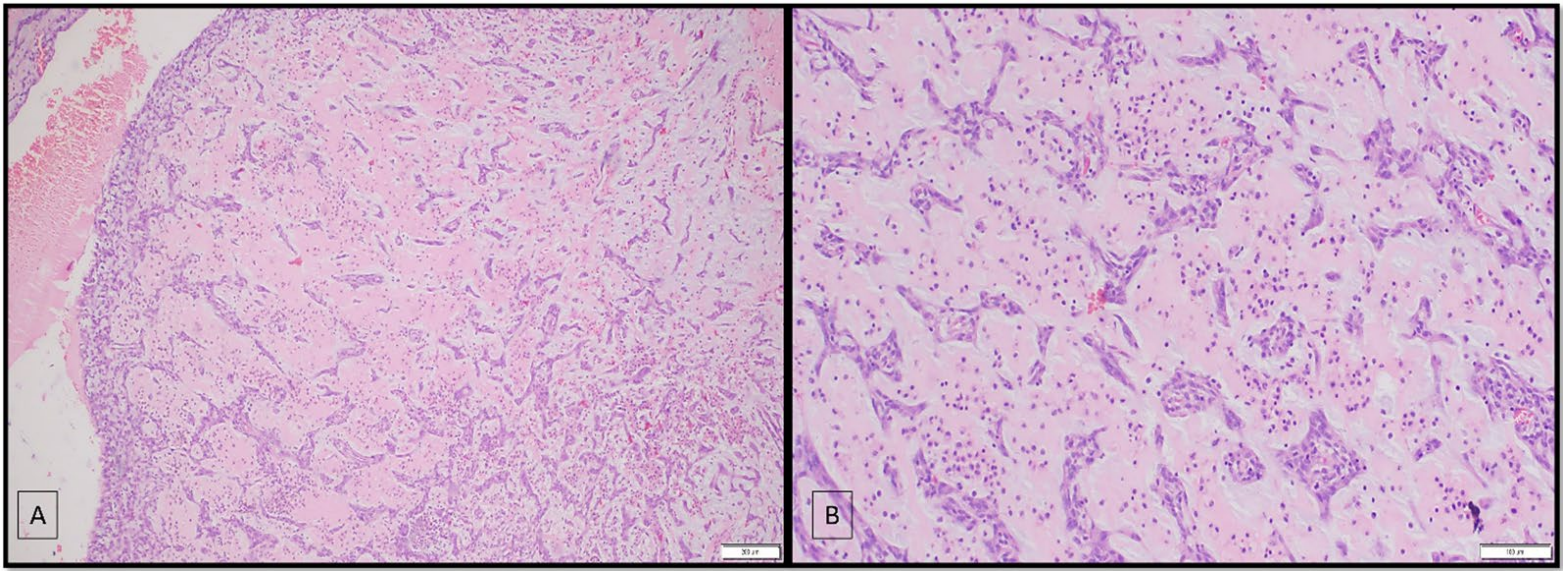
Cardiac myxoma: (**A**) Microphotography shows a tumor composed of neoplastic cells arranged in cords and nests in a branching pseudo-vascular pattern. (**B**) Higher magnification reveals polygonal-to-stellate-shaped lepidic cells in an abundant bluish myxoid background with few inflammatory cells

The patient underwent surgical excision of the mass despite the high risk of thromboembolism. Histopathology of excised specimen showed tumor composed of neoplastic cells arranged in cords and nests in a branching pseudo-vascular pattern. Higher-magnification images revealed the presence of polygonal-to-stellate-shaped lepidic cells in myxoid background. These features were compatible with diagnosis of cardiac myxoma with secondary degeneration (Fig. 4). The patient was stable in postoperative period and was discharged one week later. She was advised to take oral antiplatelet therapy (Aspirin, 325 mg once daily). The patient did not report any recurrence of symptoms and underwent a follow-up echocardiography after 1 month of surgery, which ruled out any residual or recurrent lesion.

## Conclusion

Cardiac myxomas are the most common primary cardiac neoplasms presenting as an endocardial mass occupying cardiac chambers. In majority of cases, myxomas are seen attached to the IAS and less frequently seen in ventricles. Around 75% of myxomas are seen in left atrium and 20% seen in right atrium. Cardiac myxomas usually present with obstructive constitutional symptoms such as fever, chills and lethargy, and in around 20% patients, cardiac myxomas produce no symptoms. However, in 30–40% patients, cardiac myxomas present with embolic phenomenon with clinical manifestation according to the location of the mass, the left or right side of heart. Left-sided myxomas may present with fatal cardiovascular embolic events such as stroke and coronary embolization or with complete obstruction of mitral or aortic valve. It is imperative that a diagnosis of cardiac myxoma be made using advanced imaging techniques such as echocardiography, CTA or CMR [2, 3].

Typically, cardiac myxomas present as solid mass lesions with heterogeneous enhancement on contrast administration. Cardiac myxomas can have a heterogenous appearance reflecting hemorrhage, necrosis, fibrosis or calcification and pose challenges in diagnosis and treatment. An intracardiac cystic mass with heterogenous post-contrast enhancement can have a wide list of differential diagnosis such as hydatid cyst, IAS aneurysm, coronary artery aneurysm, intramural hematoma of LA, sarcomas, hemangiomas, hemangioendotheliomas, bronchogenic cysts and rarely myxomas. It is difficult to establish a firm diagnosis due to overlapping imaging features; hence, histopathological examination is the way forward for further management and prognosis of the patient. It is necessary to adapt an aggressive diagnostic approach in case of a cystic cardiac mass to unmask stealth malignant lesions such as sarcomas which can present as benign appearing mass lesions on imaging [11–15].

This case illustrates a rare cystic form of atrial myxoma and highlights the importance of considering this condition when diagnosing apparently cystic intracardiac mass. It underscores the significance of CMR in evaluating these masses and the value of imaging techniques in distinguishing myxomas from other potential causes of intracardiac masses. Due to their structural similarities, accurately diagnosing LA cystic masses using preoperative imaging is challenging. Failure to remove these masses may lead to major complications such as stroke, rupture, and hemodynamic alterations. Therefore, surgical excision of a cystic LA mass is justified to establish a definitive diagnosis and prevent potential complications.

## Supplementary Information

Supplementary Material 1: Clip 1. CINE steady-state free precession (SSFP) horizontal long axis showing mobile cystic left atrial mass prolapsing across the mitral valve.

Supplementary Material 2: Clip 2. Dynamic first pass perfusion shows intense progressive enhancement within the cystic part of the lesion with non-enhancing hypointense solid component.

## Data Availability

Data supporting the study results can be provided followed by request sent to the corresponding author’s e-mail.

## Abbreviations

CMR: Cardiac magnetic resonance imaging
CTA: Computed tomography angiography
IAS: Interatrial septum
LA: Left atrium
LGE: Late gadolinium enhancement
LV: Left ventricle
T1WI: T1-weighted imaging
T2WI: T2-weighted imaging
